# 'It gives you an understanding you can't get from any book.' The relationship between medical students' and doctors' personal illness experiences and their performance: a qualitative and quantitative study

**DOI:** 10.1186/1472-6920-7-50

**Published:** 2007-12-05

**Authors:** Katherine Woolf, Judith Cave, I Chris McManus, Jane E Dacre

**Affiliations:** 1 Academic Centre for Medical Education, Royal Free and University College Medical School, 4th Floor, Holborn Union Building, Archway Campus, Highgate Hill, London N19 5LW, UK; 2 Dept of Psychology, University College London, Gower Street, London WC1E 6BT, UK

## Abstract

**Background:**

Anecdotes abound about doctors' personal illness experiences and the effect they have on their empathy and care of patients. We formally investigated the relationship between doctors' and medical students' personal illness experiences, their examination results, preparedness for clinical practice, learning and professional attitudes and behaviour towards patients.

**Methods:**

Newly-qualified UK doctors in 2005 (n = 2062/4784), and two cohorts of students at one London medical school (n = 640/749) participated in the quantitative arm of the study. 37 Consultants, 1 Specialist Registrar, 2 Clinical Skills Tutors and 25 newly-qualified doctors participated in the qualitative arm. Newly-qualified doctors and medical students reported their personal illness experiences in a questionnaire. Doctors' experiences were correlated with self-reported preparedness for their new clinical jobs. Students' experiences were correlated with their examination results, and self-reported anxiety and depression. Interviews with clinical teachers, newly-qualified doctors and senior doctors qualitatively investigated how personal illness experiences affect learning, professional attitudes, and behaviour.

**Results:**

85.5% of newly-qualified doctors and 54.4% of medical students reported personal illness experiences. Newly-qualified doctors who had been ill felt less prepared for starting work (p < 0.001), but those who had only experienced illness in a relative or friend felt more prepared (p = 0.02). Clinical medical students who had been ill were more anxious (p = 0.01) and had lower examination scores (p = 0.006). Doctors felt their personal illness experiences helped them empathise and communicate with patients. Medical students with more life experience were perceived as more mature, empathetic, and better learners; but illness at medical school was recognised to impede learning.

**Conclusion:**

The majority of the medical students and newly qualified doctors we studied reported personal illness experiences, and these experiences were associated with lower undergraduate examination results, higher anxiety, and lower preparedness. However reflection on such experiences may have improved professional attitudes such as empathy and compassion for patients. Future research is warranted in this area.

## Background

Adam Smith (1723–1790), the great moral philosopher and economist, recognised the importance of our own negative experiences in our ability to empathise with others:

"We have learned from experience that misfortune naturally excites such a degree of sorrow, and we know that if we took time to consider his situation, fully and in all its parts, we should, without doubt, most sincerely sympathize with him."

Adam Smith – The Theory of the Moral Sentiments [1]

Doctors experience high levels of stress and mental illness at many stages of their careers [2-7], which can negatively impact on their care of patients [8,9] and their examination performance [10]. Little published research exists, however, regarding doctors' and medical students' experiences of *physical *illness [11], although there is evidence that doctors in the United States live longer than individuals from other high socioeconomic groups, such as lawyers [12]. We found two studies which quantified students' experiences of physical illness. In one of these, 90% of 1,027 US students reported seeking healthcare during their time at medical school (including 50% for cold or 'flu, 12% for injury and 2% for cancer) [13]. In the other, 25% of 2114 US medical students reported having experienced 'personal injury or illness', and 16% 'death of a family member', in the previous year. Further, of the students with personal illness experience, those classed as 'resilient' (i.e. who did not find negative life events stressful) performed better in exams compared to those classed as 'frail' [14]. We also found one UK study which found that personal illness experience influences some medical students' career choices [15].

Doctors' personal illness experiences are related to their clinical practice, although the effect seems not to be that large. For example, results from a 1993/4 survey of all women doctors in the United States (n = 4265) showed that all the primary care physicians in the sample who had personal history of skin cancer (n = 141), but not all of those who did not, reported counselling or screening their patients for skin cancer prevention [16]. Data from the same survey indicated that doctors with a personal history of obesity, hypertension or osteoporosis were more likely to counsel their patients for weight, blood pressure or HRT (hormone replacement therapy) respectively [17]. Commenting on the relationship between personal illness experience and clinical practice, Harrison and Sterland [11] state:

'it is interesting, although not surprising, that interviews with doctor-patients reveal a common belief that encounters with personal illness strengthened them as doctors' (p11)

While this seems intuitively true, we have found little published research regarding medics' opinions of how or why their personal experiences influence their attitudes or behaviour. Our search revealed two qualitative studies about medical students' and doctors' personal illness experiences [18,19]. In the first, medical students who chose to reflect on their personal illness experiences felt this improved their empathy and respect for patients [18] – both important aspects of medical professionalism [20]. In the second, doctors' self-reported 'personal growth stories' were analysed to the conclusion that 'powerful experiences', followed by reflection and introspection, may lead to 'improved connectedness with others', or 'increased productivity, energy or creativity' [19].

More common are anecdotes, often published as 'fillers', in which healthcare professionals relate how their personal illness experiences improved their empathy, compassion and care for patients. For example a surgeon recounted how having a knee arthroplasty made him realise the emotional stress it entailed [21]; a nurse told how being diagnosed with Hepatitis C had given her a more 'compassionate outlook' towards patients [22]; a paediatric resident discussed how his own baby's ill health changed his approach to patients: 'today I meet parents of a critically ill child in our unit in a different way. I know their feelings' [23]; and a neurologist described how he self-administered botox, arguing 'it might be a good idea, where practical, for doctors to get a taste of our own medicine and experience the procedures to which we subject our patients with nary a thought for the pain or discomfort it may cause' [24].

Doctors also report how personal illness experiences affect their professional values and career choices: a physician reported that having her mother hospitalised 'brought into focus for me lessons about medical professionalism and related issues that we in medicine face. When the patient *really is your mother*, these lessons sink in fast [25];' and family doctor described how her mother's battle with cancer had lead to her own involvement in palliative care [26]. In another article, a medical student related how being hospitalised helped her reflect on her professional attitude: 'this morning, as I wait for my physicians, I think about the occasions on which I hit the snooze button one more time, reasoning that I could spend a few minutes less with each patient. I silently hope that my physicians are somehow different, more sensitive, more insightful [27].'

This study aimed to formally investigate medical students' and doctors' personal illness experiences, and specifically to:

▪ Report the frequency of personal illness experiences in a sample of doctors and medical students.

▪ Measure the relationship between personal illness experiences, and medical students' examination results and newly-qualified doctors' preparedness for their clinical jobs.

▪ Qualitatively analyse how medical students', and junior and senior doctors' personal experiences of illness affect their learning and patient care.

## Methods

### Study design

In 2005, KW gave all 1st (n = 363) and 3rd year (first year clinical) (n = 386) students at one London medical school a questionnaire – adapted from Andrews & Wilding [28] – asking them whether they had experienced 'personal serious illness, injury or assault'; 'serious illness, injury, assault in a close relative', 'death of a partner, parent or child', 'death of a close friend or relative' in the previous 3 years. They also completed the Hospital Anxiety and Depression Scale (HADS [29]), which is widely used in clinical practice and research and shows acceptable reliability [30]. The HADS contains depression and anxiety subscales, each scored from 0 to 21. Normative scores drawn from the general UK adult population are 6.14 (standard deviation = 3.76) for anxiety and 3.68 (SD = 3.07) for depression [30].

In 2005, JC gave 4784 newly qualified doctors in the UK a questionnaire asking 'have you had a serious illness in your lifetime' and 'have you had a relative or friend who has had a serious illness'. It also asked how prepared they felt by medical school for starting their first clinical posts (methods reported elsewhere [31]).

In 2004 and 2005, KW interviewed 25 clinical teachers at one London medical school, asking them what made some clinical students learn more than others (in the context of a qualitative study about the factors that affect how students learn in clinical situations). JC interviewed 25 newly qualified doctors and 15 senior doctors asking them how their experiences outside their formal training had helped them professionally (in the context of a qualitative study about looking after patients with cancer). See Table 1 for details of participants and design.

**Table 1:** Number of participants who took part in the study, and the methods used to study each group. Sex data missing for n = 8 first year students and n = 2 third year medical students

**Participant group**	**n = **	**Female**	**Male**	**Method**	**Research tool**
First year medical students	303	154	141	Quantitative	Questionnaire
Third year medical students	335	196	137	Quantitative	Questionnaire
Newly qualified doctors	2062	1214	848	Quantitative	Questionnaire
Newly qualified doctors	25	16	9	Qualitative	Semi-structured interview
Clinical teachers of medical students (18 Consultants, 5 General Practitioners, 2 Clinical Skills Tutors)	25	15	10	Qualitative	Semi-structured interview
Senior doctors (14 Consultants, 1 Specialist Registrar)	15	4	11	Qualitative	Semi-structured interview

### Ethical approval

Ethical approval for KW's study was granted by the UCL Graduate School Ethics Committee (ref: 0511/001). Questionnaire respondents gave their informed consent in writing, and interviewees gave oral informed consent. JC's study received ethical approval from Huntingdon Research Ethics Committee (questionnaire study, ref: 04/Q0104/110) and the South West Research Ethics Committee (interview study, ref: 05/MRE06/13). Questionnaire responses were considered to represent consent, and interviewees gave written consent.

### Quantitative analysis

KW calculated the relationship between medical students' examination results, HADS scores and their personal experiences using independent t-tests and nonparametric Spearman's Rho and Mann Whitney U tests. JC calculated the relationship between newly-qualified doctors' preparedness for starting work and their personal experiences of illness using Chi-squared tests and linear regression. We regarded p values less than 0.05 as significant. All statistics were calculated using SPSS for Windows v11.

### Qualitative analysis

KW and JC tape recorded and transcribed all interviews. They identified the emergent themes using the constant comparative method, paying particular attention to deviant cases, and coded the transcripts separately using Atlas.ti software. They read over the coded data together to discuss and resolve discrepancies where possible.

## Results

84.6% (307/363) of first year students, 86.3% (333/386) of third year students and 43% (n = 2062/4784) of newly-qualified doctors responded to the questionnaires.

### Frequency of personal illness experiences

15.8% (48/303) of first year medical students and 18.5% (61/330) of third year medical students reported serious illness, injury or assault in the previous three years; 38.8% (118/303) of first year students and 35.6% (117/330) of third year students reported injury, illness, assault or death in a close friend or relative in the previous three years.

5.9% (121/2051) of newly qualified doctors reported a serious illness in their lifetime and 84.7% (1742/2062) reported a serious illness in a close friend or relative.

### Personal illness experience, exam results and preparedness

Third year medical students who had experienced personal ill health in the previous three years had lower examination results than those who had not (t = 2.8; df = 321; p = 0.006). The effect size was *d *= 0.31, which Cohen considers of medium order [32] {the effect size was calculated using the effect size calculator at [33], which uses the following formula: *d *= M1 - M2/σpooled; where σpooled = √[(σ1^2 ^+ σ2^2^)/2], where M1 and M2 are the means for each group (in this case, those who had experienced the event, and those who had not), and σ1 and σ2 are the standard deviations}. There was a non-significant trend for higher exam scores the further away in time the personal illness experience occurred.

**Figure 1:**
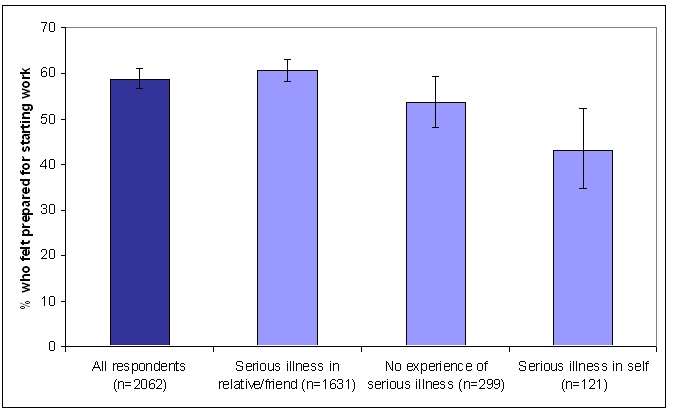


Significantly fewer doctors who had suffered a serious illness in their lifetime felt prepared for starting work (χ2 = 12.4; df = 1; p < 0.001) (Figure 1). For the doctors who had never been ill themselves, illness in a close friend or relative was associated with higher preparedness (61% vs 54%; χ2 = 5.3; df = 1; p = 0.02).

### Personal experiences, anxiety and depression

Mean anxiety (1st year: 5.60, SD = 3.25; 3rd year: 6.16, SD = 3.37) and depression (1st year: 2.83, SD = 2.2; 3rd year: 2.68, SD = 2.27) scores were similar for both student cohorts (median depression scores: 1st year = 2; 3rd year = 2). Anxiety scores were approximately normally distributed. The depression score distributions for both cohorts appeared to be censored at the bottom end and therefore non-parametric tests were used to analyse those data.

**Figure 2:**
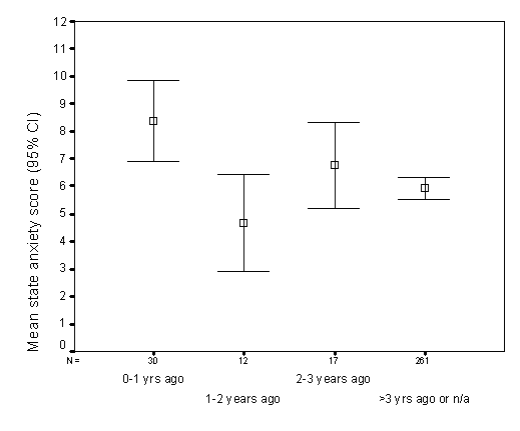


Third, but not first, year students reported serious illness, injury or assault were more anxious than those who did not (t = 2.54; df = 318; p = 0.012). The effect size was small to medium at *d *= 0.29. Students who reported serious illness, injury or assault in the previous year were statistically significantly more anxious than those who had had such an experience more than three years previously or not at all (z = -3.48; p = 0.001; *d *= 0.45;), or 1–2 years previously (z = -2.89 p = 0.003; *d *= 0.93). There were only 12 students in this last group, and therefore caution should be taken in the interpretation of this result (Figure 2).

Third year students who reported illness in a relative or friend were higher on depression (z = -2.17; p = 0.03). There was no such correlation in first year students (z = -1.87; p = 0.06).

### Anxiety, depression and examination results

**Figure 3:**
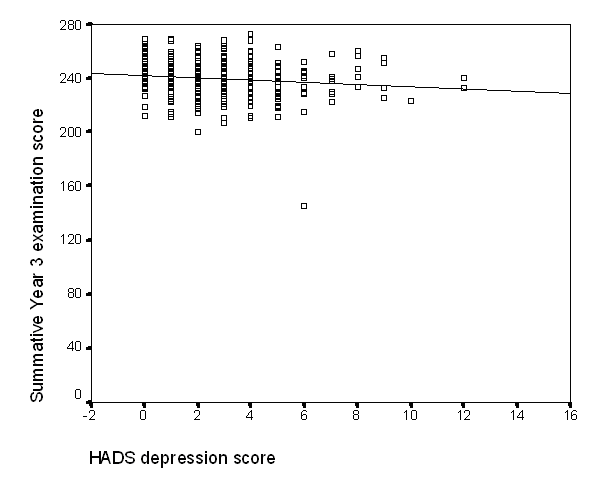


There was a weak negative correlation between depression score and 3rd, but not 1st, year examination score (Spearman's Rho = -0.13; p = 0.019) – see Figure 3. Anxiety scores were not related to 1st (r = 0.027; p = 0.64) or 3rd year examination results (r = 0.03; p = 0.65).

### Interview results

Clinical teachers considered 'life experience' a valuable asset for medical students, which they equated with maturity, being a better learner, and more empathetic.

*KW: "What might make some students learn more or less than each other in a clinical setting?"* "I would say one word and the word is maturity. If the student has an understanding of life and people then medicine is more interesting. If you understand about people and life it's a damned site easier to take in knowledge from your patients and that's what medicine should be about." [Teacher 2, male, Consultant Physician]

"A key aspect of being a good medical student is maturity and insight."*KW: [...]" can you explain a little bit more about what maturity might mean?"*" Maturity is [...] dealing with sensitive issues, you know, personal problems of patients – some students are more immediately sensitive to and comfortable with that than others [...] I guess being able to put yourself in other people's shoes." [Teacher 5, male, Consultant Physician]

"A sprinkling of graduate students helps learning: they have more life experience, what the GMC [General Medical Council] would call 'transferable skills'." [Teacher 23, male, Consultant Physician]

Clinical teachers recognised, however, that students' personal experiences of illness could impede learning.

**"**If there's an emotional problem at home, father dying or whatever, that gets in the way of the learning in their own time." [Teacher 13, male Consultant Physician]

"I've seen students in the welfare clinic who have longstanding financial or health-related problems of their own, or family issues and er, they're going to impinge on their studies." [Teacher 17, female, Consultant Physician]

One clinical teacher thought personal illness experiences could motivate students.

"If [the students] have got any personal or family experience of that illness or a related illness, they're often very strongly motivated because they've had a family member with a similar disease." [Teacher 8, Consultant Surgeon]

Newly qualified doctors felt their personal illness experiences had made them more patient or relative centred.

*JC****:****"What do you think it was about your training that helped to prepare you for looking after these patients?"* "It's not my training. It's personal experience. [...]. My dad has multiple myeloma; um, my mum had high, er, high grade Non-Hodgkin's lymphoma [...]. You know what they [the patients] will be thinking." [Junior doctor 18]

**"**My Mum had breast cancer [...] [It] makes me a bit nicer to [the patients]. It makes me think 'well hang on a minute, I'm, I may be a bit hungry because I haven't had breakfast you know, I haven't eaten today, but this poor woman is dying [...] put her first." [Junior doctor 19]

They also learnt about implications of clinical practice and procedures.

"One of my family members has been sort of going through the whole colonoscopy-type investigation thing [...]. When it's like somebody that you know and they have to get through the whole er bowel prep thing and it's pretty disgusting and there's actually not...it's not as routine, most of the investigations, as you think they are until you see somebody do it. So I guess I wouldn't order a colonoscopy now unless they actually really need it [laughs]." [Junior doctor 5]

"My Nan developed a pleural effusion [...] they were like 'ooh she's only got a few weeks to live'. Well that was two years ago' [...] 'I would never ever, ever ever tell someone that I thought there was something big and serious going on until I knew more." [Junior doctor 25]

One interviewee described how a personal experience had hindered her training, making her distance herself from clinical experiences to avoid becoming upset.

"I think [my grandparents dying from cancer] makes it more difficult than actually helps [...]. I'm dreadful with death [...] I hate listening to people give people diagnoses [...] particularly cancer diagnoses for some reason and I sit and look shut off and I stand in the background." [Junior doctor 22]

Similarly, senior doctors described personal experiences which had moved them profoundly, and had helped them relate to patients and see their viewpoint.

"I came to my own hospital with my daughter before I was a Consultant here and [...] all the issues that you regard as being important as a healthcare professional become very irrelevant when you're a patient or a parent of a patient. It's very humbling actually to, to be put on the other side of the river." [Senior doctor 3, male]

"Personal experience of true grief and bereavement gives you an understanding that you can't get from any theoretical book on the grieving process." [Senior doctor 7, male]

## Discussion

The majority of doctors (85.5%) and medical students (54.4%) in our samples reported personal illness experiences. Personal illness was correlated with lower examination results and higher anxiety in clinical medical students, and with lower preparedness in newly-qualified doctors. Experience of illness in a close relative was correlated with higher depression in clinical medical students; however, newly-qualified doctors who had never been seriously ill themselves but had experienced illness in a relative or friend felt *more *prepared. Clinical teachers perceived students with life experience and maturity as more insightful and better learners, although they also recognised that illness could compromise learning as it occurred. Newly-qualified and senior doctors felt their personal illness experiences made them more empathic and patient or relative-centred.

This study is one of the first to describe the frequency of both doctors' and medical students' personal illness experiences, the first to investigate the relationship between personal experiences and preparedness in junior doctors, and the first to qualitatively study how doctors feel their personal experiences affect their professional attitudes and behaviour. Our survey of all newly-qualified doctors in 2005 enabled a powerful statistical analysis of the relationship between personal illness experiences and preparedness. The qualitative data provided an insight into the relationship between personal illness experience and learning in medical students, and personal illness experience and clinical practice of junior and senior doctors.

This article combines data from two separate studies, which did not ask exactly the same questions. However, we felt that the relative dearth of evidence justified this combining of data to explore personal experiences at many stages of doctors' careers. It is rare to find survey studies with 100% response rates, and the percentage of junior doctors who responded to our survey (43%) compares reasonably well to other studies of junior doctors in the UK [34-36]. As with most surveys, it is useful to consider possible differences between respondents and non-respondents. In this case, the questions we asked junior doctors were mostly concerned with preparedness for starting work, rather than about personal illness experiences, and it therefore seems unlikely that non-respondents were put off responding to the questionnaire by the subject. However, it is worth bearing in mind that more depressed, anxious or stressed individuals may not have responded, and it is conceivable that those individuals may have experienced more negative life events than the respondents.

Even bearing in mind the high response rate from students (~85%) the study could have been improved by increasing the number of medical students surveyed: the relatively small number who reported illness experiences possibly prevented us from finding statistically significant results. Furthermore, the data are cross-sectional, and therefore provide a basis for future longitudinal research, which would allow causality to be inferred.

The percentage of medical students in our cohorts who reported illness, injury or assault in the previous three years (17.2%) was higher than the percentage of newly qualified doctors who said they have had a serious illness in their lifetime (5.9%). Perhaps junior doctors, who spend most of their time caring for patients with illnesses severe enough to warrant hospital admission, were less likely to define an illness as 'serious'. It may also be that the doctors did not think of 'assault' and 'injury' as illnesses per se. Conversely, many more doctors (84.7%) reported having experienced illness in close friends or relatives compared to medical students (37.2%). This may be because the medical students reported events from the previous three years, whereas doctors reported events from throughout their lives. Moreover, junior doctors are generally older than medical students, and hence more of their family will have succumbed to mortality. Additionally, perhaps relatives and friends were more likely to discuss their illnesses with qualified doctors than with medical students. Reported rates of personal illness experiences in our cohorts are low in comparison with the rates reported in Hojat et al.' study[14], possibly because of slight differences in the questions. Further qualification of the term "serious" in the questionnaire could reduce possible differences in its interpretation.

The anxiety and depression scores in our sample of medical students were comparable with normative data from the general population [37] and with data from medicine and non-medicine undergraduate students [38]. Being a medical student is stressful [39] so it is reassuring that our cohorts had relatively low anxiety and depression. Andrews and Wilding [28] found personal illness in non-medicine undergraduates caused depression, which decreased examination marks. Our results similarly show that third year students who experienced illness in a relative or friend were more likely to be depressed, and depression was significantly negatively correlated with examination results. The relationship between anxiety and examination performance is less clear. In our study, students who reported personal illness were more anxious and did worse in their examinations, but the level of anxiety was not related to the level of decrease in examination scores. Similar non-causal relationships between these variables have been shown previously [28,39]. It may be that the medical students who reported higher levels of state anxiety were also higher on the personality trait *neuroticism *[40]. High *neuroticism *individuals tend to be more anxious [41] and report more illness symptoms [41]. Perhaps students who reported being ill were not more anxious because they were ill, but remembered more illness and were more anxious because they were higher on *neuroticism*. Indeed, students in Hojat et al's study who had experienced a negative event (including financial and academic problems) performed lower on some examinations, were more anxious and higher on *neuroticism *compared to those who had not [14].

The newly-qualified doctors who had been seriously ill felt less prepared, by medical school, for their clinical jobs. If those illnesses had occurred during their medical school careers, they may have had fewer learning opportunities, which may have affected their preparedness. If those doctors had learned less, they might be expected to have performed less well in their medical school examinations. While this information is not known for our cohort of doctors, we do know that clinical medical students who reported illness, injury or assault performed lower in examinations than those who had not. It is interesting that newly-qualified doctors who reported a illness in a close friend or relative felt more prepared. One could speculate that those personal experiences did not result in a loss of learning opportunities in the way that personal illness might, and instead provided benefits – possibly the improved empathy and patient-centeredness described in the qualitative data.

Our qualitative data corroborate anecdotes that personal illness experience improves empathy and patient-centeredness, and also support the two qualitative studies from the literature [18,19]. Furthermore, that the clinical teachers felt that students who had had more life experience and were more mature were more empathetic and better learners is supported by a study that found older medical students differed from younger students on their understanding of the role of a doctor, as well as on various other measures relating to learning [42]. Kern *et al*. [19] suggest that reflecting on personal experiences can be the first step in making new meanings from material or experiences, so-called 'transformational learning'[43] – indeed, the anecdotes reported in the Introduction above were written by doctors who, by definition, had reflected on their personal experiences.

## Conclusion

This study provides evidence that the majority of medical students in our sample and newly-qualified doctors will have at least one illness experience, either themselves or in a close friend or relative. The quantitative evidence indicates that personal illness can be detrimental to learning in the short term, and is related to higher levels of anxiety and depression – important information for medical educators and those responsible for student welfare. The qualitative evidence provides a silver lining for a rather gloomy cloud suggesting that personal experiences of illness may ultimately improve doctors' empathy, patient-centeredness, and professional behaviour, possibly via reflection and transformational learning processes. Enabling doctors to reflect on their personal experiences may increase their positive effects, although this should be undertaken with caution.

## Competing interests

The author(s) declare that they have no competing interests.

## Authors' contributions

KW and JC conceived of the idea for the article. KW and ICM designed the medical student questionnaire, and KW and JED administered it. JED provided the medical student examination data. JC designed the junior doctor questionnaire with input from KW and JED; it was administered by JC and KW. JC and KW performed the data analysis. KW and JC wrote the first draft of the paper, and ICM contributed to subsequent drafts. All authors approved the final version of the manuscript.

## Pre-publication history

The pre-publication history for this paper can be accessed here:


